# Emergence of West African Human T-Lymphotropic Virus 1aC Subgroup, Brazilian Amazon

**DOI:** 10.3201/eid3207.260372

**Published:** 2026-07

**Authors:** Jean de Melo Silva, Emmily Myrella Vasconcelos Mourão, Enzo Miranda Santos, Luma Silva Mineiro, Paulo Henrique Rodrigues de Souza, Leonardo Calheiros de Oliveira, Jacqueline da Silva Batista, Giselle Moura Guimarães Marques, Carolina Rosadas de Oliveira, Graham P. Taylor, Antonio Carlos Rosário Vallinoto, Gemilson Soares Pontes

**Affiliations:** Institute of Biological Science, Federal University of Amazonas, Manaus, Brazil (J. de Melo Silva, G.S. Pontes); Leônidas and Maria Deane Institute–ILMD/Fiocruz Amazônia, Manaus (E.M.V. Mourão); Foundation of Hematology and Hemotherapy of Amazonas, Manaus (E.M. Santos); National Institute of Amazonian Research, Manaus (L.S. Mineiro, P.H.R. de Souza, L.C. de Oliveira, J. da Silva Batista, G.M. Guimarães Marques, G.S. Pontes); Faculty of Medicine, Imperial College London, London, UK (C.R. de Oliveira, G.P. Taylor); National Centre for Human Retrovirology, St. Mary's Hospital, Imperial College Healthcare NHS Trust, London (G.P. Taylor); Federal University of Pará, Belém, Brazil (A.C.R. Vallinoto)

**Keywords:** viruses, human T-lymphotropic virus, HTLV-1aC, phylogeography, genomic surveillance, molecular epidemiology, Brazilian Amazon, Brazil

## Abstract

In a cross-sectional survey of 1,397 residents of Manaus, Brazil, we found a seroprevalence of 0.3% for human T-lymphotropic viruses (HTLVs) 1/2 and identified HTLV type 1aC by phylogenetic analysis. Those findings provide evidence of introduction of West African HTLV-1aC into the Brazilian Amazon and highlight regional limitations in genomic surveillance.

Epidemiologic surveillance is essential for defining the burden, geographic distribution, and transmission patterns of human T-lymphotropic viruses (HTLVs) 1 and 2 (HTLV-1/2), particularly in underserved settings such as the Brazilian Amazon. Although Brazil is estimated to harbor the largest absolute number of HTLV-1 infections worldwide, data from urban Amazonian populations remain scarce (*1*,*2*). In this context, molecular surveillance is also critical because HTLV genetic diversity is geographically structured, could have clinical relevance, and helps refine understanding of lineage distribution and viral dissemination (*3*). We therefore investigated HTLV-1/2 infection in the metropolitan region of Manaus in the Amazonas state of Brazil and performed phylogenetic and phylogeographic analyses.

During May 2021–November 2023, we conducted a cross-sectional study of 1,397 residents of metropolitan Manaus recruited by convenience sampling across different city zones at universities, community centers, polyclinics, and primary healthcare units. Eligible participants were Manaus residents >7 years of age who voluntarily agreed to participate and provided written informed consent; for minors, we obtained parental or guardian consent and assent when applicable. We screened plasma samples for HTLV-1/2 antibodies by ELISA and confirmed seroreactive samples by Western blot or line immunoassay; indeterminate samples underwent molecular testing. We collected sociodemographic and behavioral data through a standardized questionnaire. 

We amplified the 5′ long terminal repeat (LTR) region of HTLV-1 (579 bp) and HTLV-2 (788 bp) by nested PCR and sequenced the amplicons by Sanger sequencing. We inferred phylogenetic relationships by maximum-likelihood analysis in MEGA12 (https://www.megasoftware.net) using the Kimura 2-parameter model with 1,000 bootstrap replicates and a geographically diverse panel of HTLV-1 and HTLV-2 reference sequences retrieved from GenBank from a previously assembled dataset. We deposited sequences generated in this study into GenBank (accession nos. PV647910 for sequence WDM1168_BrMao, PV647909 for sequence WQC1199_BrMao, PV647908 for sequence SSS850_BrMao, and PV742404 for sequence JDC1001_BrMao). To investigate the timing and geographic history of HTLV-1 lineages, we performed Bayesian time-scaled phylogenetic and discrete phylogeographic analyses in BEAST X version 10.5 (https://beast.community).

Overall HTLV-1/2 seroprevalence was 0.3% (95% CI 0.11%–0.73%; 4/1,397 participants), comprising 3 HTLV-1 and 1 HTLV-2 infections. All seropositive participants were >40 years of age and shared a profile of socioeconomic vulnerability: 75% were female, earned no more than minimum wage, had only elementary education, lacked marital partnerships, and relied on government assistance (Appendix). Phylogenetic analysis classified sequence SSS850_BrMao as HTLV-1aC, the West African subgroup, providing evidence of this lineage in the Brazilian Amazon (Figure 1, panel A). The remaining isolates were HTLV-1aA (WDM1168_BrMao and WQC1199_BrMao) and HTLV-2c (JDC1001_BrMao), indicating co-circulation of distinct HTLV lineages in this population (Figure 1).

**Figure 1:**
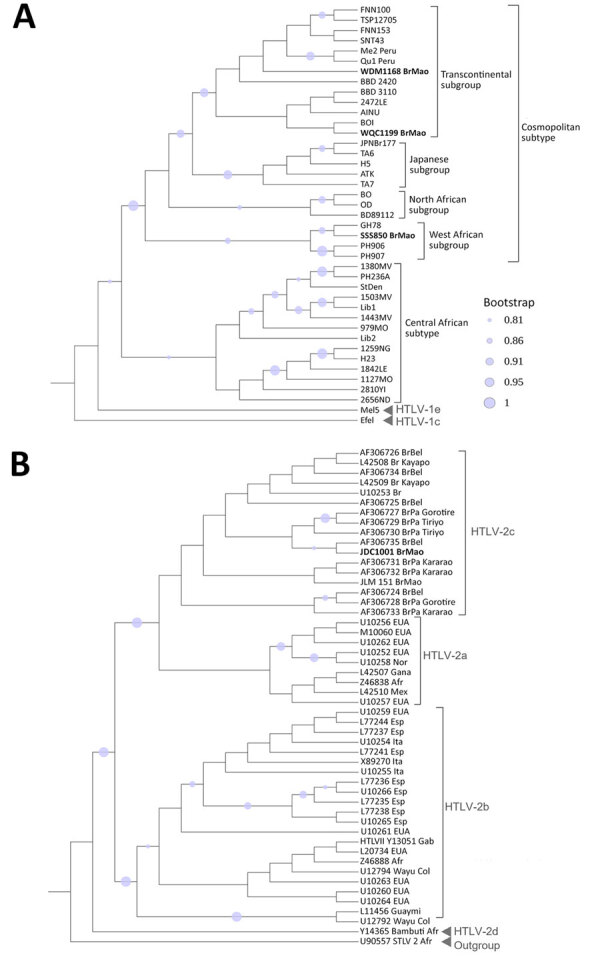
Phylogenetic tree of HTLV isolates in study of emergence of West African HTLV-1aC subgroup, Brazilian Amazon. A) HTLV-1 sequences obtained in this study (bold text) were analyzed with 38 reference sequences from GenBank. The Manaus isolates clustered within the Cosmopolitan subtype, including the Transcontinental subgroup (WDM1168_BrMao and WQC1199_BrMao) and the West African subgroup (SSS850_BrMao). B) The HTLV-2 sequence identified in Manaus (JDC1001_BrMao, bold text) was analyzed with 49 reference sequences from GenBank (accession numbers provided) and clustered within subtype HTLV-2c. Trees were inferred using the maximum-likelihood method under the Kimura 2-parameter substitution model. The best-scoring trees (log-likelihood −14,610.1 for panel A and −2,247.45 for panel B) were selected through heuristic searches initiated with neighbor-joining and maximum parsimony starting topologies. Branch support was assessed with 1,000 bootstrap replicates. HTLV, human T-lymphotropic virus.

After we identified the HTLV-1aC lineage, we performed Bayesian time-scaled phylogenetic and discrete phylogeographic analyses to infer its introduction and evolutionary history (Figure 2). Our results demonstrate that strain SSS850_BrMao clusters robustly within the HTLV-1aC clade, supporting its introduction into Amazonas around 2015 without evidence of intermediate dispersal through Pará. In contrast, 2 HTLV-1aA sequences clustered with reference sequences from Japan, indicating a separate introduction into northern Brazil via Pará (≈1971) followed by spread to Amazonas (≈1996). Together, those findings reveal >2 subtype-stratified introductions and identify HTLV-1aC as an epidemiologically relevant lineage in the region. However, because the phylogeographic inferences were based on a ≈579-bp 5′ LTR region fragment, they should be interpreted cautiously.

**Figure 2:**
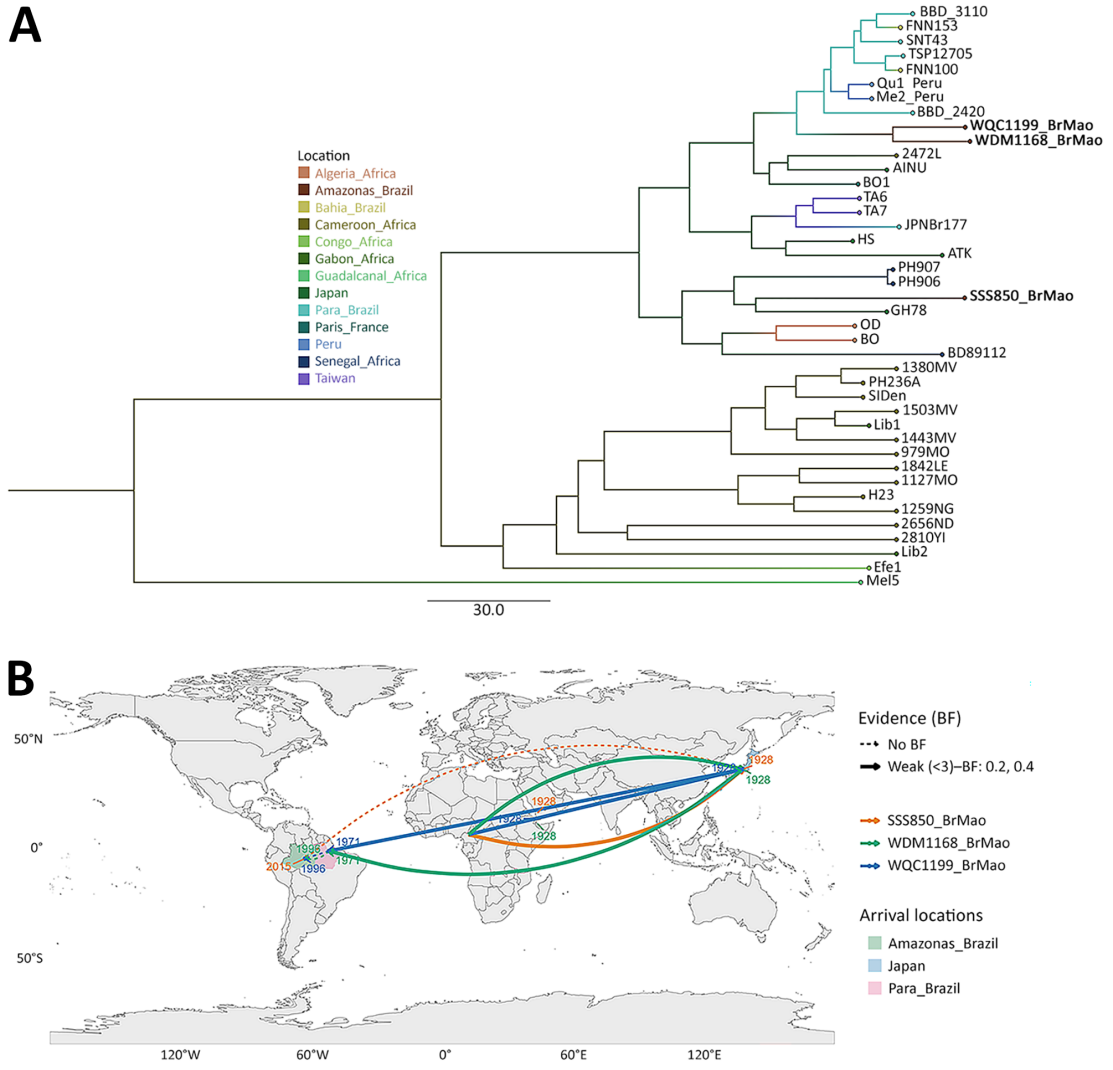
Time-scaled phylogeny and discrete phylogeographic diffusion of HTLV-1 lineages in study of emergence of West African HTLV-1aC subgroup, Brazilian Amazon. A) Time-calibrated maximum clade credibility tree inferred in BEAST version 10.5 (https://beast.community) from the long terminal repeat (LTR) dataset (3 sequences from this study plus reference sequences), using sampling year as tip date, a strict molecular clock, and discrete geographic-state reconstruction under a continuous-time Markov chain model with Bayesian stochastic search variable selection. Branches and tips are colored by inferred location (see key); bold text indicates sequences generated in this study. B) Spatiotemporal diffusion reconstructed from Markov jumps. Trajectory colors correspond to each Manaus lineage: orange, SSS850_BrMao; green, WDM1168_BrMao; blue, WQC1199_BrMao. Numbers indicate posterior median year of first arrival (labels shown for years >1970; Cameroon→Japan step, ≈1928, is retained for context). Line style indicates support: dashed, no Bayers factor recovered; solid, weak support (<3; here, 0.2–0.4). Shaded polygons indicate inferred arrival locations (Amazonas and Pará states, Brazil, and Japan). HTLV, human T-lymphotropic virus.

We observed a HTLV-1/2 prevalence in metropolitan Manaus, lower than estimates from other regions of Brazil, including Salvador and Mato Grosso do Sul (*4*,*5*). However, comparisons should be made with caution because sampling strategies and population composition differ across studies. Beyond prevalence, the key finding was detection of HTLV-1aC. Although this subgroup has previously been reported in Brazil by restriction fragment length polymorphism analysis (*6*), our study provides phylogenetically supported evidence of HTLV-1aC’s occurrence in the country and places it in phylogeographic context. This result broadens the known distribution of HTLV-1aC and suggests that viral diversity in northern Brazil may be greater than previously recognized, despite evidence that HTLV-1aA predominates nationwide (*7*,*8*). Phylogeographic analyses supported >2 subgroup-specific introductions into the region, highlighting HTLV-1aC as an epidemiologically relevant lineage. We also identified HTLV-2c in an urban resident, reinforcing evidence that this subtype is not restricted to Indigenous populations and might circulate across multiple epidemiologic settings in northern Brazil (*9*,*10*).

The first limitation of our study is that we used convenience sampling at multiple sites, rather than a population-based design. Therefore, the sample might not fully represent the city’s general population. The small number of positive participants precluded robust inference on risk factors or transmission routes. Phylogeographic reconstruction was further constrained by use of a short 5′ LTR fragment from a slowly evolving virus and by uneven regional sequence availability, reducing temporal and geographic resolution.

Despite those limitations, detection of HTLV-1aC in Manaus has surveillance relevance because rare lineages might remain unnoticed in settings with limited molecular monitoring. Broader epidemiologic surveillance combined with longer genomic regions and regionally representative sampling will be essential to refine lineage dispersion patterns, strengthen transmission hypotheses, and support HTLV prevention, diagnosis, and care policies tailored to the Brazilian Amazon.

Appendix Additional information about emergence of West African human T-lymphotropic virus 1aC subgroup, Brazilian Amazon.
