# Supplementary material for: Emergence of West African Human T-Lymphotropic Virus 1aC Subgroup, Brazilian Amazon
Source: Emerg Infect Dis. 2026 Jul;32(7):1207–11. doi: 10.3201/eid3207.260372 (PMC13322426; doi:10.3201/eid3207.260372)
Supplement: Appendix — Additional information about emergence of West African human T-lymphotropic virus 1aC subgroup, Brazilian Amazon. [file 26-0372-Techapp-s1.pdf]

Article DOI: https://doi.org/10.3201/eid3207.260372

*EID cannot ensure accessibility for supplementary materials supplied by authors. Readers who have difficulty accessing supplementary content should contact the authors for assistance.*

# Emergence of West African Human T-Lymphotropic Virus 1aC Subgroup, Brazilian Amazon

## Appendix

**Appendix Table.** Sociodemographic and behavioral characteristics of seropositive individuals

| | Participant ID | | | |
|---|---|---|---|---|
| Variables | SSS850 | WDM1168 | WQC1199 | JDC1001 |
| Age (years) | 46 | 45 | 63 | 68 |
| Sex | Female | Female | Female | Male |
| Race/Ethnicity (self-reported) | Mixed-race | Mixed-race | Mixed-race | Black |
| Country of birth | Brazil | Brazil | Brazil | Brazil |
| State of birth | Pará | Amazonas | Amazonas | Amazonas |
| Length of residence in Manaus | >7 y | >7 y | >7 y | >7 y |
| Marital status | Married/Living together | Single | Single | Widowed |
| Education | High school completed | Elementary school completed | Elementary school incomplete | Elementary school incomplete |
| Family household income (minimum wage) | 1 | <1 | <1 | 2 |
| government assistance | Yes | Yes | Yes | Yes |
| Number of pregnancies | 3 | 4 | 6 | Not applicable |
| Breastfeeding duration | >6 mo | >6 mo | >6 mo | Not applicable |
| Age at sexual debut | 18 y | 18 y | 14 y | Don't remember |
| Number of sexual partners in the past 12 mo | 1 | 1 | No response | 1 |
| History of STI diagnosis | No | No | No | No |
| Tattoo | No | Yes | No | No |
| History of blood Transfusion | No | No | Yes | Yes |
| Breastfed in infancy | Yes | Yes | Yes | Yes |
| Condom Use | Yes | Yes | No | No |

Abbreviations: STI, sexually transmitted infection.
